# Inactivation of the *Rcan2* Gene in Mice Ameliorates the Age- and Diet-Induced Obesity by Causing a Reduction in Food Intake

**DOI:** 10.1371/journal.pone.0014605

**Published:** 2011-01-27

**Authors:** Xiao-yang Sun, Yoshitaka Hayashi, Sai Xu, Yasuhiko Kanou, Yoshiko Takagishi, Ya-ping Tang, Yoshiharu Murata

**Affiliations:** 1 Division of Stress Adaptation and Recognition, Department of Genetics, Research Institute of Environmental Medicine, Nagoya University, Nagoya, Japan; 2 Department of Cell Biology and Anatomy, Louisiana State University Health Sciences Center, New Orleans, Louisiana, United States of America; Institute of Preventive Medicine, Denmark

## Abstract

Obesity is a serious international health problem that increases the risk of several diet-related chronic diseases. The genetic factors predisposing to obesity are little understood. *Rcan2* was originally identified as a thyroid hormone-responsive gene. In the mouse, two splicing variants that harbor distinct tissue-specific expression patterns have been identified: *Rcan2-3* is expressed predominately in the brain, whereas *Rcan2-1* is expressed in the brain and other tissues such as the heart and skeletal muscle. Here, we show that *Rcan2* plays an important role in the development of age- and diet-induced obesity. We found that although the loss of *Rcan2* function in mice slowed growth in the first few weeks after birth, it also significantly ameliorated age- and diet-induced obesity in the mice by causing a reduction in food intake rather than increased energy expenditure. *Rcan2* expression was most prominent in the ventromedial, dorsomedial and paraventricular hypothalamic nuclei governing energy balance. Fasting and refeeding experiment showed that only *Rcan2-3* mRNA expression is up-regulated in the hypothalamus by fasting, and loss of *Rcan2* significantly attenuates the hyperphagic response to starvation. Using double-mutant (*Lep^ob/ob^ Rcan2* ^−/−^) mice, we were also able to demonstrate that *Rcan2* and leptin regulate body weight through different pathways. Our findings indicate that there may be an *Rcan2*-dependent mechanism which regulates food intake and promotes weight gain through a leptin-independent pathway. This study provides novel information on the control of body weight in mice and should improve our understanding of the mechanisms of obesity in humans.

## Introduction

Obesity has reached epidemic proportions globally over the past several decades, and now poses a major risk for serious diet-related chronic diseases, such as type 2 diabetes and cardiovascular diseases [1]–[3]. It is widely accepted that obesity is a result of the interaction of genetic and environmental factors [4]. Epidemiological evidence has clearly shown direct linkage between overnutrition and obesity [5], [6]. In rodents, high-fat diets also have been demonstrated to induce obesity [7], [8]. However, the genetic factors linking obesity to overnutrion are poorly understood.

*RCAN2* (also known as *ZAKI*-4, *Dscr1l1*, *MCIP2* or *Calcipressin* 2) [9] was originally identified as a thyroid hormone (T3)-responsive gene in cultured human skin fibroblasts [10] and subsequently reported to function as a regulator of calcineurin [11], [12]. In the mouse, two splicing variants that harbor distinct tissue-specific expression patterns have been identified: *Rcan2-3* (formerly named *ZAKI-4α*) is expressed predominately in the brain, whereas *Rcan2-1* (formerly named *ZAKI-4β*) is expressed in the brain and other tissues such as the heart and skeletal muscle [13].

To determine the function of *Rcan2* in the whole organism, we generated *Rcan2*-null (*Rcan2* ^−/−^) mice by targeted disruption of the exon 4 which is used by *Rcan2-1* and *Rcan2-3*. We fortuitously found that *Rcan2* ^−/−^ mice showed reduced body weight and white adipose mass compared to the control mice, especially, they showed a significantly reduced rate of weight gain on the high-fat diet. We analyzed food intake, energy expenditure and expression of genes involved in the control of energy metabolism. The results showed that *Rcan2* regulates food intake and body weight through the mechanism independent from leptin pathway. These findings provide novel insights into the mechanisms of body weight regulation and should have important implications to studies on obesity in human populations.

## Results and Discussion

**Figure 1:**
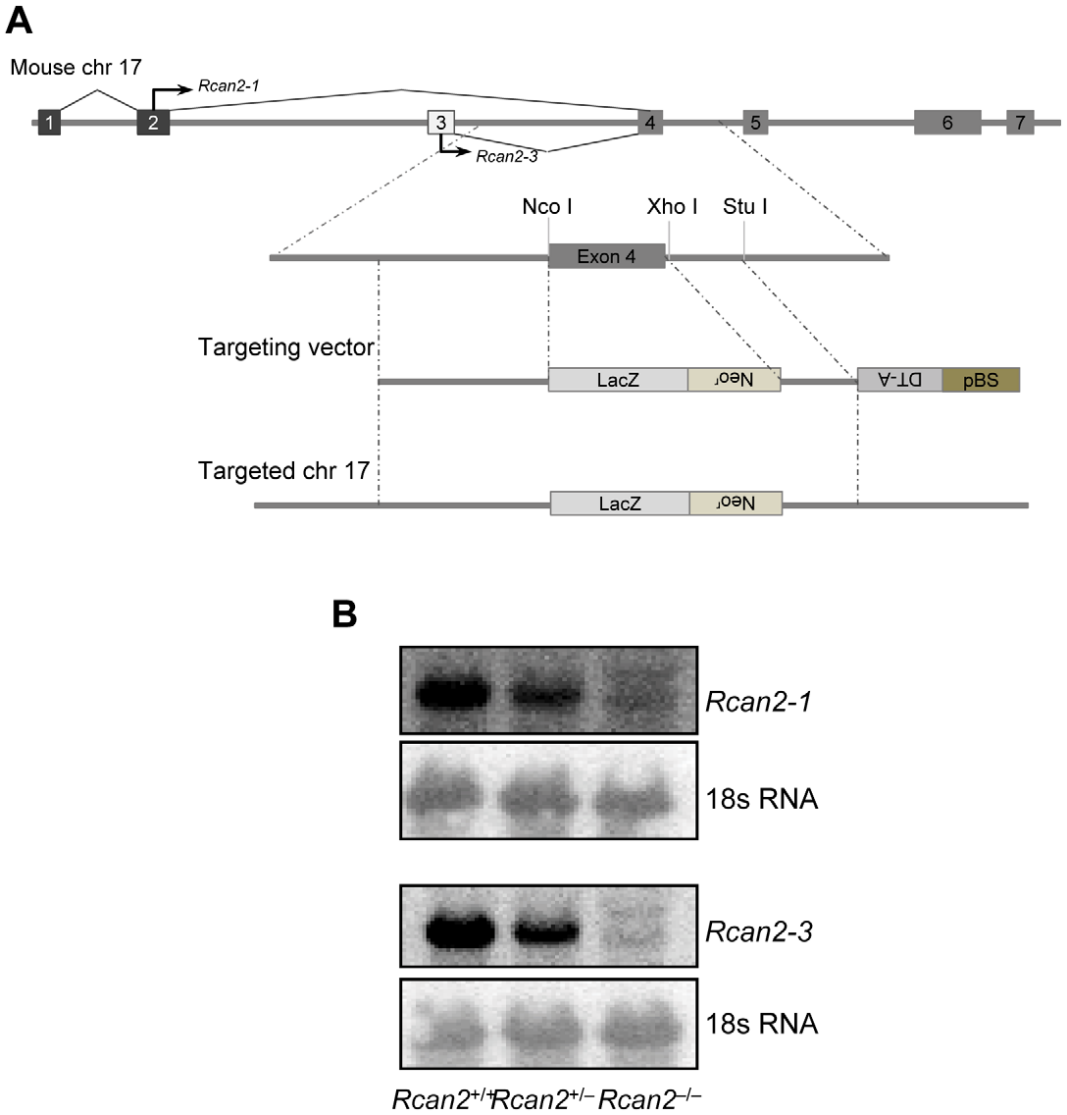
Generation of *Rcan2*-deficient mice. (A) Schematic description of the strategy used to generate the mutant *Rcan2* allele. LacZ, promoter-less LacZ gene; Neo^r^, neomycin resistance gene; DT-A, diphtheria toxin gene; pBS, pBluescript II vector. (B) Representative Northern blots. Brain total RNA was probed with *Rcan2-1* or *Rcan2-3* specific sequences.

To evaluate the physiological role of *Rcan2*, we generated *Rcan2* ^−/−^ mice in which a *LacZ/Neo* cassette replaced exon 4 (Figure 1A). Northern blotting analyses confirmed the absence of *Rcan2-1* and *Rcan2-3* in *Rcan2* ^−/−^ mice (Figure 1B). Although an earlier study did not report any obvious differences between *Rcan2* ^−/−^ mice and their littermates on a 129Sv/B6 mixed genetic background [14], we found that on the B6 background alone, *Rcan2* ^−/−^ mice had significantly lower body weights than their littermates.

**Figure 2:**
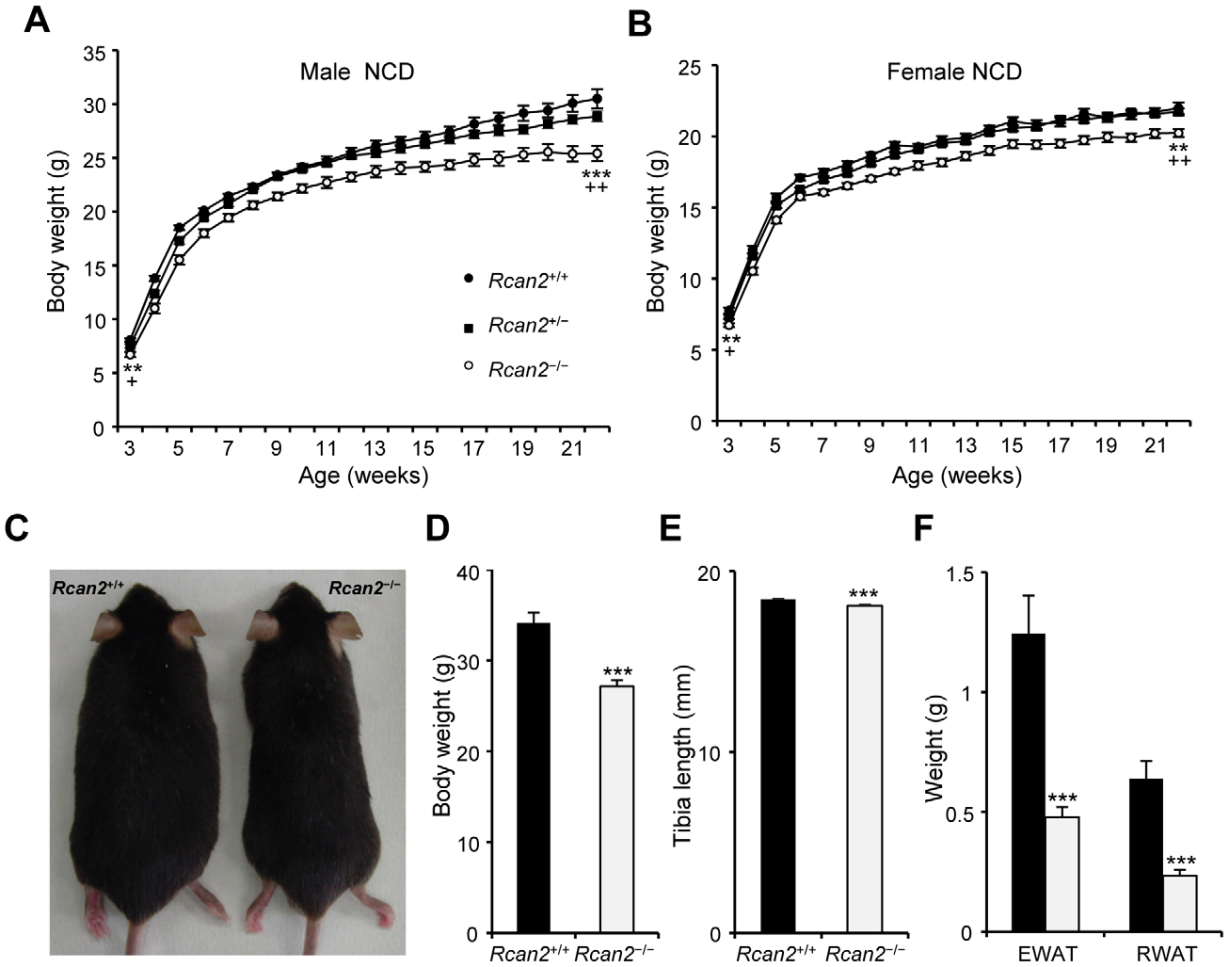
Reduced body weights in *Rcan2* ^−/−^ mice. (A, B) Growth curves of male (A) and female (B) mice fed a normal chow diet. Sixteen *Rcan2* ^+/+^, 29 *Rcan2* ^+/−^, and 15 *Rcan2* ^−/−^ males were analyzed from postnatal week 3 to week 10, and 9, 16, and 7, respectively, after postnatal week 10. Eighteen *Rcan2* ^+/+^, 34 *Rcan2* ^+/−^, and 23 *Rcan2* ^−/−^ females were analyzed from postnatal week 3 to week 10, and 11, 19, and 17, respectively, after postnatal week 10. **: P<0.001, ***: P<0.0001 (vs *Rcan2* ^+/+^); +: P<0.05, ++: P<0.005 (vs *Rcan2* ^+/−^). (C) Representative examples of 9-month-old *Rcan2* ^+/+^ and *Rcan2* ^−/−^ males. (D) Mean body weights of ten 9-month-old *Rcan2* ^+/+^ and *Rcan2* ^−/−^ males. (E) Mean tibia lengths of eight 9-month-old *Rcan2* ^+/+^ and *Rcan2* ^−/−^ males. (F) Mean weight of epididymal and retroperitoneal white adipose tissue (EWAT and RWAT, respectively) in ten 9-month-old *Rcan2* ^+/+^ and *Rcan2* ^−/−^ males. All values are given as mean ± s.e.m. ***: P<0.0001. Filled circles/columns indicate *Rcan2* ^+/+^ mice; open circles/columns, *Rcan2* ^−/−^ mice.

The postnatal weights of mice produced by intercrossing *Rcan2* ^+/−^ heterozygotes were measured. At birth, there were no significant differences between genotypes (data not shown). However, by 3 weeks of age, *Rcan2* ^−/−^ mice were smaller than their littermates and continued to show slower growth up to 5 weeks of age (Figure 2A and 2B). There was little evidence of genotype-related variation in growth rates from 5 to 10 weeks of age but, later, the body weights of *Rcan2* ^−/−^ males increased more slowly. As a consequence, at 22 weeks of age, *Rcan2* ^−/−^ males weighed about 16.7% less than wild-type males (25.4±0.7 g in *Rcan2* ^−/−^ mice versus 30.5±0.9 g in wild type; p<0.0001) (Figure 2A). By comparison, *Rcan2* ^−/−^ females were only 8% smaller than their wild-type littermates at 22 weeks of age (20.2±0.3 g in *Rcan2* ^−/−^ mice versus 22.0±0.4 g in wild type; p<0.001) (Figure 2B). Nine-month-old *Rcan2* ^−/−^ males were approximately 20% smaller than their wild-type littermates (27.2±0.7 g in *Rcan2* ^−/−^ mice versus 34.1±1.1 g in wild type; p<0.0001) (Figure 2C and 2D). Measurement of tibia lengths showed those of *Rcan2* ^−/−^ mice were only 2% shorter than the wild-type (18.09±0.06 mm in *Rcan2* ^−/−^ mice versus 18.45±0.03 mm in wild type; p<0.0001) (Figure 2E). However, white adipose tissues (WAT) were markedly reduced in *Rcan2* ^−/−^ mice at about 60% of the wild-type (Figure 2F). The weight differences continually increased as the animals aged. At 18 months of age, *Rcan2* ^−/−^ males weighed about 27.7% less than wild-type males (31.6±1.1 g in *Rcan2* ^−/−^ mice versus 43.7±1.4 g in wild type; p<0.0001, n = 6 in each group) (data not shown). These data indicate that loss of Rcan2 function slowed the rate of growth in the initial weeks after birth, and significantly ameliorated, at least in male mice, age-related obesity.

**Figure 3:**
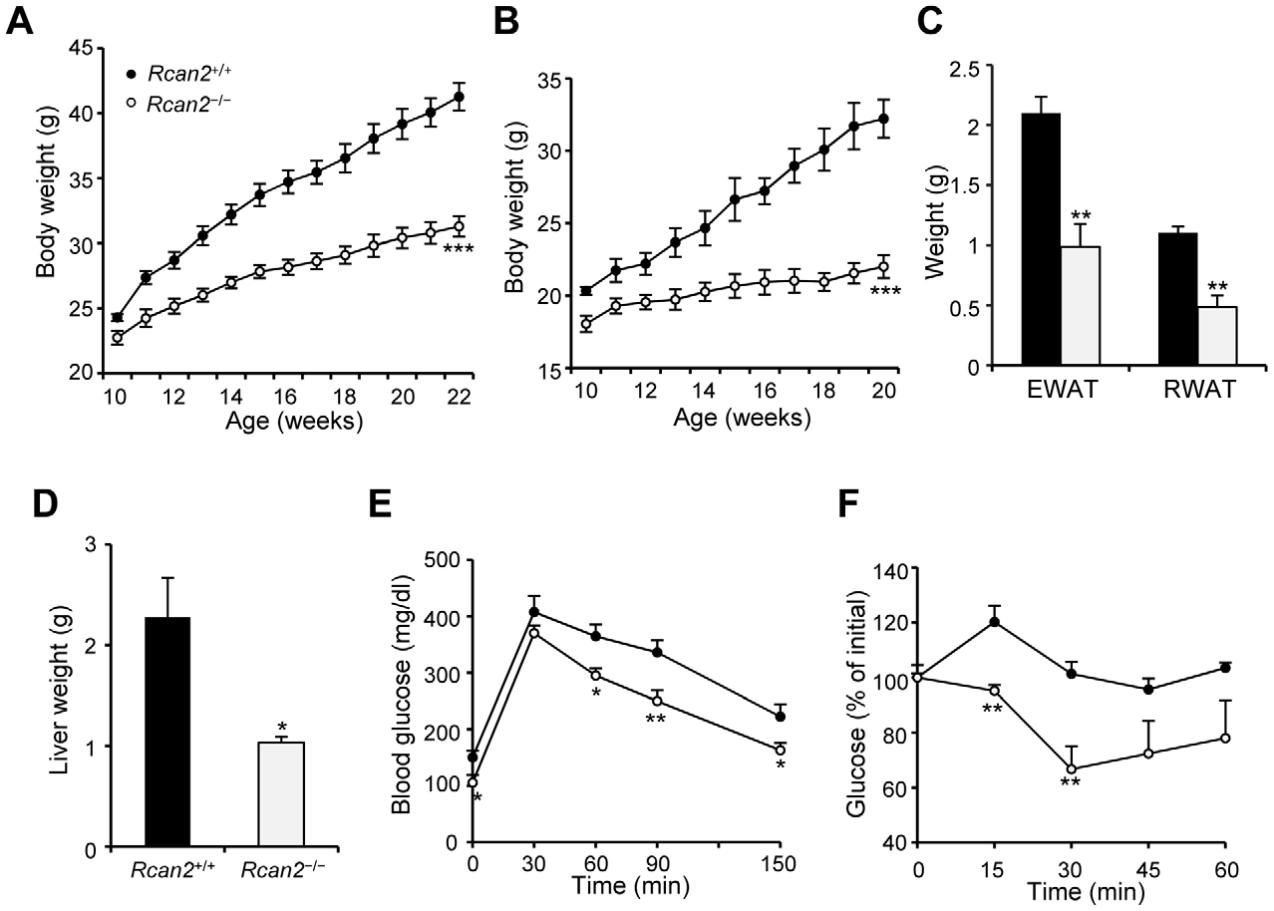
Amelioration of high-fat diet-induced obesity in *Rcan2* ^−/−^ mice. (A, B) Growth curves of 9 *Rcan2* ^+/+^ and 10 *Rcan2* ^−/−^ males (A) and 5 *Rcan2* ^+/+^ and 5 *Rcan2* ^−/−^ females (B) mice fed on a high-fat diet for 12 weeks. ***: P<0.0001. (C) Mean weight of epididymal and retroperitoneal white adipose tissue (EWAT and RWAT, respectively) in four *Rcan2* ^+/+^ and *Rcan2* ^−/−^ males fed on a high-fat diet for 12 weeks. (D) Mean weight of liver in four *Rcan2* ^+/+^ and *Rcan2* ^−/−^ males fed on a high-fat diet for 12 weeks. (E) Glucose tolerance test. n = 6 in each group. (F) Insulin tolerance test. n = 6 in each group. All values are given as mean ± s.e.m. *: P<0.05; **: P<0.01. Filled circles/columns indicate *Rcan2* ^+/+^ mice; open circles/columns, *Rcan2* ^−/−^ mice.

Since *Rcan2* ^−/−^ mice gained less weight on the normal chow diet, we sought to determine their response to diet-induced obesity. When *Rcan2* ^−/−^ mice were challenged with a high-fat diet from 10 weeks of age, they showed a significantly reduced rate of weight accumulation over a 12-week period compared to their wild-type littermates (Figure 3A and 3B). At the end of the experiment, *Rcan2* ^−/−^ males weighed an average of 10 g less than wild-type males (31.3±0.8 g in *Rcan2* ^−/−^ mice versus 41.3±1.1 g in wild type; p<0.0001) (Figure 3A), compared to 5.1 g on the normal chow diet (25.4±0.7 g in *Rcan2* ^−/−^ mice versus 30.5±0.9 g in wild type; p<0.0001) (Figure 2A). The difference in response to the high-fat diet was also pronounced in female mice (Figure 3B). The *Rcan2* ^−/−^ mice had significantly reduced WAT weights after the high-fat diet compared to wild-type mice (Figure 3C). Also, livers of *Rcan2* ^−/−^ mice weighed on average approximately 1.2 g less than those of wild-type mice (Figure 3D) and did not exhibit the typical feature of fatty liver observed in wild-type mice (data not shown). Because diet-induced obesity always causes insulin resistance and glucose intolerance, we also evaluated whether *Rcan2* ^−/−^ mice showed any changes among them. An intraperitoneal glucose tolerance test showed that *Rcan2* ^−/−^ mice can clear glucose more efficiently than wild-type controls (Figure 3E). When mice were intraperitoneally injected with a fixed dose of insulin, *Rcan2* ^−/−^ mice showed a much more prolonged and pronounced hypoglycemia than wild-type mice, indicating an increase of insulin sensitivity (Figure 3F).

Recently, it has been demonstrated that *Rps6kb1* ^−/−^ mice manifested slow growth in the first several weeks after birth [15], and were resistant to age- and high-fat diet induced obesity [16]. These phenotypes are similar to those of *Rcan2* ^−/−^ mice. *Rps6kb1* ^−/−^ mice appear to be protected against obesity due to the up-regulation of genes such as uncoupling protein 1 (*Ucp1*), *Ucp3*, Ppar-γ co-activator-α (*Pgc1*-α) and peroxisome proliferator-activated receptor β/δ (*Ppar*-β/δ) that control the oxidative phosphorylation pathway [16]. To explore whether a similar pattern of gene up-regulation is responsible for the *Rcan2* ^−/−^ phenotype, we analyzed the expression levels of these genes in skeletal muscle and brown adipose tissue. No significant differences were found between *Rcan2* ^−/−^ and wild-type mice (Figure S1A and S1B). These results indicate that the mechanism for protection against obesity differs between *Rcan2* ^−/−^ and *Rps6kb1* ^−/−^ mice.

**Figure 4:**
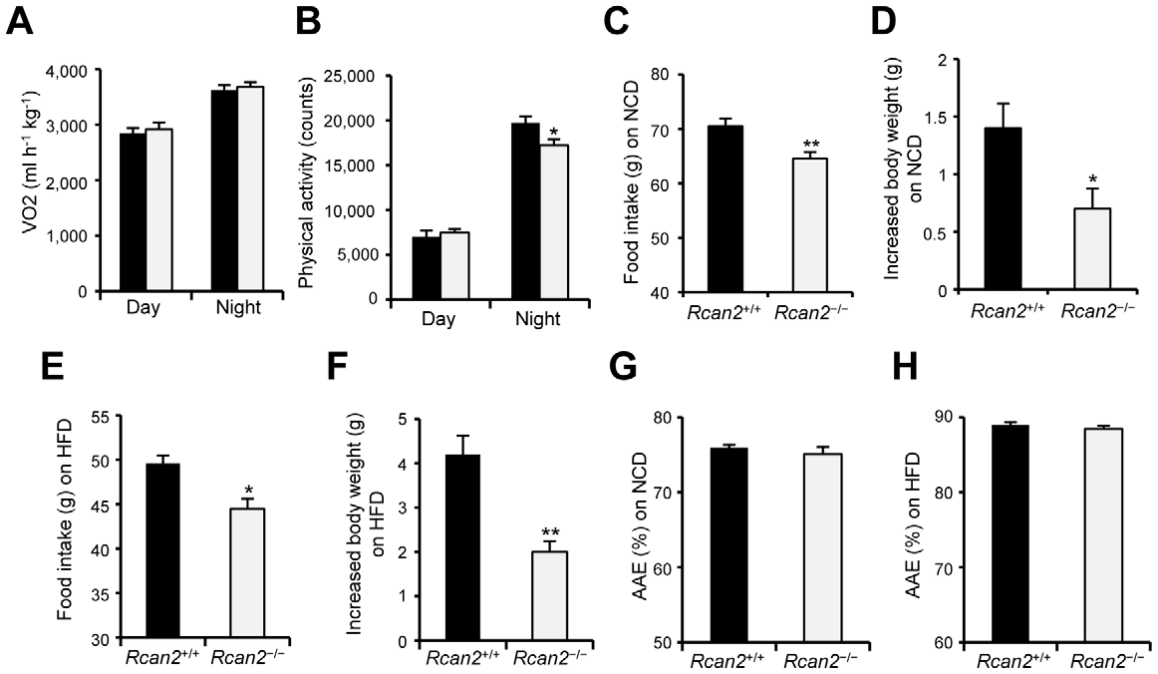
Parameters of energy expenditure and food intake. (A, B) Oxygen consumption (VO2) (A) and physical activity (B) in weight-matched 9-week-old *Rcan2* ^+/+^ and *Rcan2* ^−/−^ males fed the normal chow diet (NCD). Four males of each genotype were analyzed. (C, D) Cumulative food intake (C) and increased body weight (D) measured from postnatal week 13 to week 15 in 6 *Rcan2* ^+/+^ and 6 *Rcan2* ^−/−^ males fed the NCD. (E, F) Cumulative food intake (E) and increased body weight (F) measured from postnatal week 11 to week 13 in 3 *Rcan2* ^+/+^ and 4 *Rcan2* ^−/−^ males fed the high-fat diet (HFD). (G, H) Apparent absorption efficiency (AAE) measured in mice fed either the NCD (G) or the HFD (H). Three *Rcan2* ^+/+^ and 4 *Rcan2* ^−/−^ males were used for each experiment. All values are given as mean ± s.e.m. *: P<0.05; **: P<0.01. Filled columns indicate *Rcan2* ^+/+^ mice; open columns, *Rcan2* ^−/−^ mice.

To identify possible causes of differential body weight gain, we used the CLAMS system to measure energy expenditure. Since lean mass is the major determinant of energy expenditure [17]–[19], weight-matched 9-week-old males were selected for the analyses. No significant differences were observed in oxygen consumption (VO_2_) (Figure 4A) or carbon dioxide production (data not shown), but *Rcan2* ^−/−^ mice showed a slight decrease in cumulative physical activity during the night period (Figure 4B). These analyses indicated that *Rcan2* ^−/−^ mice had similar energy expenditure as weight-matched controls. We then monitored food intake and body weights in male mice on the normal chow diet from postnatal week 13 to week 15, the period in which differences in body weight gain between *Rcan2* ^−/−^ and wild-type male mice were prominent. During this period, *Rcan2* ^−/−^ male mice ingested about 8.5% less food (64.57±1.22 g in *Rcan2* ^−/−^ mice versus 70.54±1.41 g in wild type; p<0.005) (Figure 4C) and gained 0.7 g less body weight than wild-type controls (0.70±0.17 g in *Rcan2* ^−/−^ mice versus 1.40±0.21 g in wild type; p<0.05) (Figure 4D). Therefore, differential food intake contributes, at least in part, to the differential weight gain. Similar measurements were obtained for male mice on the high-fat diet from postnatal week 11 to week 13. During this period, *Rcan2* ^−/−^ mice ingested about 10.3% less food (44.49±1.14 g in *Rcan2* ^−/−^ mice versus 49.58±0.91 g in wild type; p<0.02) (Figure 4E) and gained 2.2 g less body weight than controls (2.00±0.24 g in *Rcan2* ^−/−^ mice versus 4.20±0.42 g in wild type; p<0.005) (Figure 4F). The similar reduction of food intake in *Rcan2* ^−/−^ mice on the normal chow or high-fat diet suggests that *Rcan2* might regulate food intake in a uniform manner regardless of its quality. We excluded the possibility of malabsorption in *Rcan2* ^−/−^ mice by calculating apparent absorption efficiency. The analysis showed that loss of *Rcan2* had no significant effect on food absorption either on the normal chow diet (75.1±1.0% in *Rcan2* ^−/−^ mice versus 76.0±0.4% in wild type; p = 0.32) (Figure 4G) or on the high-fat diet (88.5±0.4% in *Rcan2* ^−/−^ mice versus 88.9±0.4% in wild type; p = 0.38) (Figure 4H).

**Figure 5:**
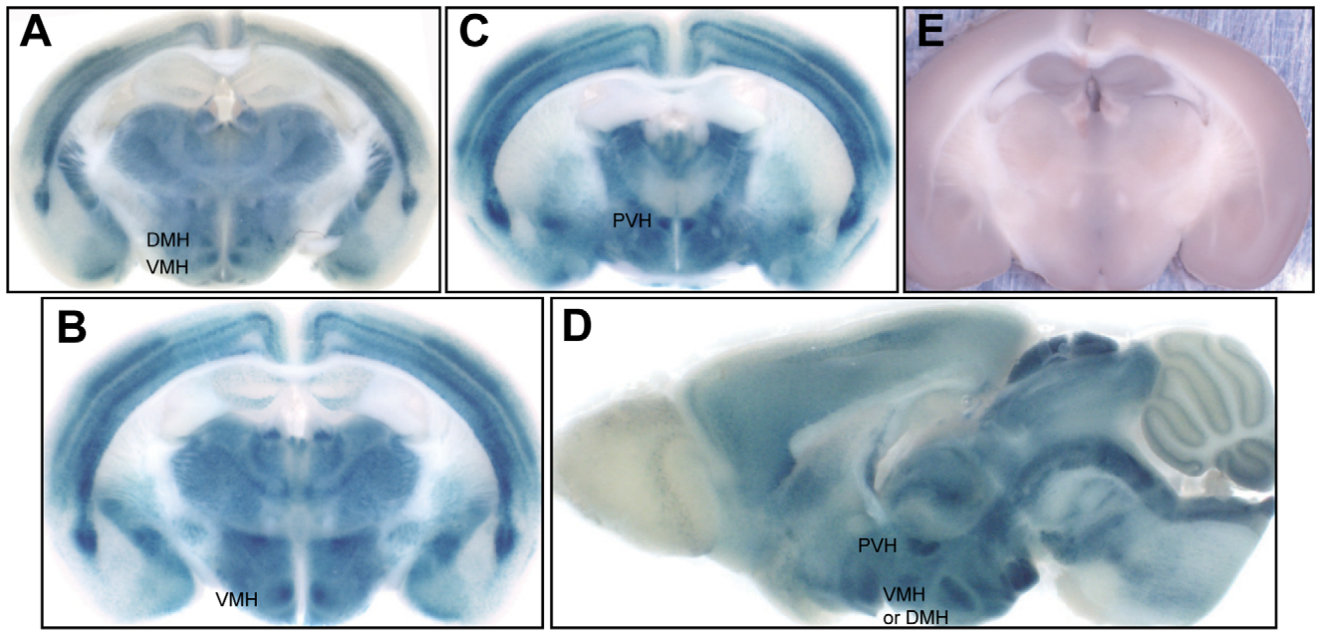
Distribution of Rcan2 in the hypothalamus. (A-D) X-gal staining of brain coronal sections (A–C) and sagittal sections (D) revealed that Rcan2 was highly expressed in the dorsomedial (DMH), ventromedial (VMH) and paraventricular (PVH) hypothalamic nuclei. (E) X-gal staining of wild-type controls.

Taken together, our data suggested that the reduced body weight of *Rcan2* ^−/−^ mice was attributable to reduced food intake. Food intake is mainly controlled by regulatory centers in the hypothalamus [20]–[22]. We examined expression of *Rcan2* in the hypothalamus by using X-gal staining. Analysis of stained sections of brain tissue showed that *Rcan2* was widely expressed, and was particularly prominent in hypothalamic nuclei such as the ventromedial (VMH), dorsomedial (DMH), and paraventricular (PVH) hypothalamic nuclei (Figure 5A–5D). Mice with lesions in the VMH and PVH show hyperphagia and obesity suggesting these regions are involved in regulation of feeding and body weight [23], [24]. Thus, the distribution patterns of *Rcan2* suggest that it might play a role in the regulation of food intake. Although *Rcan2* has been reported as a regulator of calcineurin [11], [12], its distribution in the brain did not coincide with that of calcineurin [25], which is highly expressed in the hippocampus [26], [27]. The non-overlapping distribution suggests that hypothalamic *Rcan2* may have calcineurin-unrelated functions. To address this question, we measured hypothalamic calcineurin activity in *Rcan2* ^−/−^ mice and controls and found no significant difference between the two groups (Figure S2).

**Figure 6:**
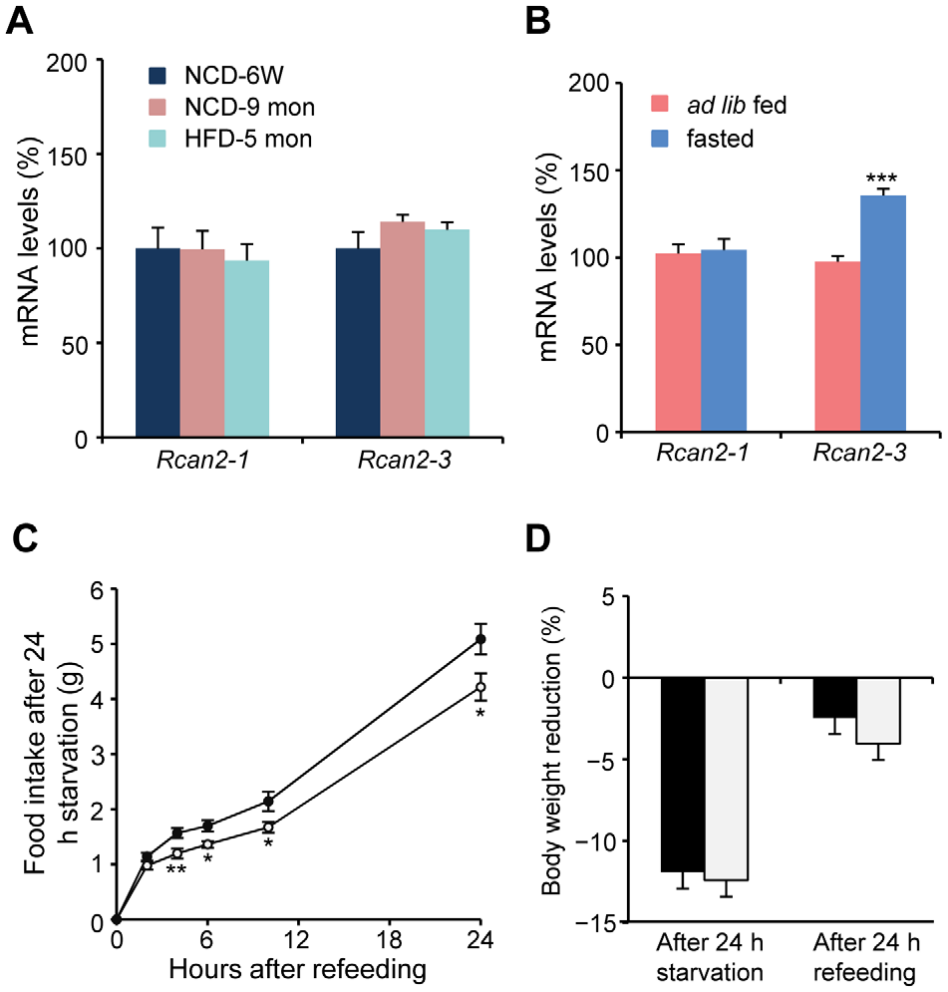
Expression of *Rcan2* mRNA in the hypothalamus and its involvement in the hyperphagic responses. (A) Expression of hypothalamic *Rcan2-1* and *Rcan2-3* mRNAs in the *ad lib* fed state. Total RNA was isolated from hypothalami of 6-week-old wild-type mice or 9-month-old wild-type mice that was *ad lib* fed with the normal chow diet (NCD), or from hypothalami of wild-type mice that had been fed with the high-fat diet (HFD) for 5 months. The RNA was used for quantitative real-time PCR analysis using primers specific for *Rcan2-1*, *Rcan2-3* and *β–actin* (internal standard). mRNA expression levels are expressed relative to those of 6-week-old mice. NCD-6W indicates 6-week-old mice fed with the NCD; NCD-9 mon, 9-month-old mice fed with the NCD; HFD-5 mon, wild-type mice fed with the HFD for 5 months. n = 5 in each group (B) Expression of hypothalamic *Rcan2-1* and *Rcan2-3* mRNAs in the fasted state. Total RNA was isolated from hypothalami of wild-type male that were allowed to feed *ad lib* or were fasted for 24 h. The RNA was used for quantitative real-time PCR analysis using primers specific for *Rcan2-1*, *Rcan2-3* and *β–actin* (internal standard). mRNA expression levels are expressed relative to those of *ad lib* fed wild-type mice. Data were expressed as the mean of three independent experiments. Thirty-one *ad lib* fed wild-type mice and 30 fasted wild-type mice were used in this analysis. Red columns indicate the mRNA expression levels in the *ad lib* fed state; blue columns, the mRNA expression levels in the fasted state. (C) Cumulative food intake in response to 24-h fasting. Six wild-type males (28.90±0.67 g) and six *Rcan2* ^−/−^ males (24.78±0.81 g) were analyzed. (D) Body weight reduction after 24-h fasting and 24-h refeeding. Six males of each genotype were analyzed. All values are given as mean ± s.e.m. *: P<0.05; **: P = 0.01; ***: P<0.0001. Filled circles/columns indicate *Rcan2* ^+/+^ mice; open circles/columns, *Rcan2* ^−/−^ mice.

Given the clear distribution of *Rcan2* in the hypothalamus, we investigated whether hypothalamic *Rcan2* mRNA expression is regulated in wild-type mice. From six weeks of age, both *Rcan2-1* and *Rcan2-3* mRNAs were expressed at a relatively constant level in the fed state as the animals aged either on the normal chow diet or on the high-fat diet (Figure 6A). However, we found that 24 hours of fasting specifically increased expression of *Rcan2-3* mRNA, the splicing variant of which is predominately expressed in the brain by about 40% in the hypothalamus (Figure 6B). Considering that mice respond to 24 hours of fasting with compensatory hyperphagia, we then examined whether the increased *Rcan2-3* expression is involved in the hyperphagic response. *Rcan2* ^−/−^ mice began to show significant difference in cumulative food intake from wild-type mice after 4-hours refeeding (p = 0.01). After 24-hours refeeding, *Rcan2* ^−/−^ mice ingested about 17.1% less food than wild-type mice (4.22±0.25 g in *Rcan2* ^−/−^ mice versus 5.09±0.28 g in wild type; p<0.05) (Figure 6C). Since in the *ad lib* fed state, *Rcan2* ^−/−^ mice ingested about 10% less food than wild-type controls (Figure 4C and 4E), these data suggest that up-regulation of *Rcan2-3* expression might be involved in the hyperphagic response to fasting. Weight loss was comparable between *Rcan2* ^−/−^ and wild-type mice after 24 hours of fasting (12.41±0.80% in *Rcan2* ^−/−^ mice versus 11.91±0.48% in wild type; p = 0.57) (Figure 6D).

We next investigated whether lack of *Rcan2* might affect expression of the hypothalamic neuropeptides proopiomelanocortin (POMC), agouti-related peptide (AgRP), neuropeptide Y (NPY), prepro-orexin, and melanin-concentrating hormone (MCH) that are considered to be regulators of feeding and energy balance [20]–[22]. Expression of these neuropeptides in the hypothalamus did not differ between *Rcan2* ^−/−^ and control mice in the fed or fasted states (Figure S3).

Currently, body weight and adipose mass are considered to be tightly regulated by homeostatic mechanisms in which leptin, an adipocyte secreted hormone [28], provides a major feedback signal to the hypothalamus [20]–[22]. Leptin circulates at levels proportional to body fat content [29], [30] and acts on hypothalamic neurons that express the neuropeptides. Leptin regulates food intake and energy expenditure through these neurons depending on the status of the adipose tissues [20]–[22]. Fasting decreases leptin levels in the body, which leads to a hyperphagic response by increasing the expression of neuropeptides (e.g. AgRP/NPY, prepro-orexin and MCH) in these neurons. Since *Rcan2-3* expression was found to be up-regulated in the hypothalamus by fasting, we investigated whether the up-regulation of *Rcan2-3* expression is caused by the low leptin levels. Previous studies showed that hypothalamic neuropeptides, such as NPY and MCH which are up-regulated by fasting, are also over-expressed in leptin-dificient (*Lep^ob/ob^*) mice [31], [32]. We then examined whether *Rcan2-3* is over-expressed in *Lep^ob/ob^* mice. No significant difference was found between *Lep^ob/ob^* and wild-type mice (Figure S4). This analysis thus indicates that *Rcan2-3* expression is not regulated by leptin.

**Figure 7:**
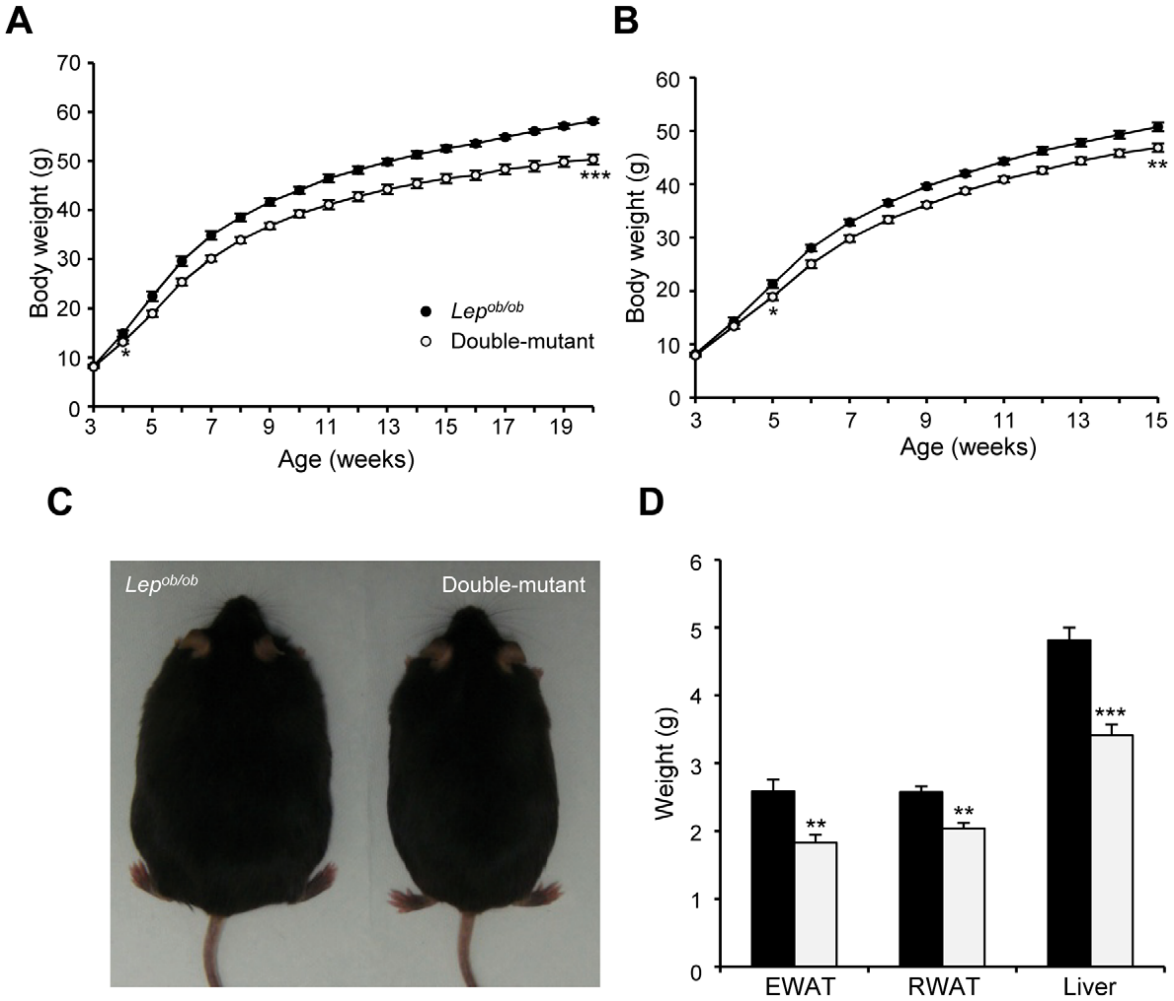
Reduced body weights in *Lep^ob/ob^ Rcan2* ^−/−^ double-mutant mice. (A, B) Growth curves of male (A) and female (B) *Lep^ob/ob^* and double-mutant mice fed the normal chow diet. Eleven *Lep^ob/ob^* and 14 double-mutant males were measured from postnatal week 3 to week 10, and 7 and 9, respectively, after postnatal week 10. Eleven *Lep^ob/ob^* and 13 double-mutant females were measured. *: P<0.05, **: P<0.01, ***: P<0.0001. (C) Appearance of 20-week-old *Lep^ob/ob^* and double-mutant male mice. (D) Weights of epididymal and retroperitoneal white adipose tissue (EWAT and RWAT, respectively) and of liver in 6 *Lep* ^ob/ob^ and 6 double-mutant males. All values are given as mean ± s.e.m. **: P<0.005; ***: P<0.0001. Filled circles/columns indicate *Lep* ^ob/ob^ mice; open circles/columns, double-mutant mice.

To further explore the possibility that *Rcan2* might regulate body weight via a leptin-independent pathway, we introduced the *Rcan2* mutation into *Lep^ob/ob^* mice. Double-mutant (*Lep^ob/ob^ Rcan2* ^−/−^) mice were generated by intercrossing doubly heterozygous mice. The weights of animals fed a normal chow diet were monitored from 3 weeks of age. Double-mutant males showed lower body weights than *Lep^ob/ob^* males from 4 weeks (Figure 7A). In females, significant differences were evident by 5 weeks (Figure 7B). At 20 weeks of age, double-mutant males weighed about 13.5% less than *Lep^ob/ob^* males (50.3±1.14 g in double-mutant mice versus 58.2±0.4 g in *Lep^ob/ob^* mice; p<0.0001) (Figure 7A and 7C). This weight reduction was reflected in decreased weights of WAT and liver in the double-mutant males (Figure 7D). In females, the weight differences between double-mutant and *Lep^ob/ob^* mice slightly increased as the animals aged (Figure 7B), similar to the growth patterns seen in *Rcan2* ^−/−^ and wild-type females fed the normal chow diet (Figure 2B). Moreover, as shown in Table S1, when we analyzed how the loss of *Rcan2* (or leptin) affected body weight by calculating the ratios of body weights of age-matched mice (Data from Figure 2A, 2B, 7A and 7B), we found that absence of *Rcan2* on the wild-type or *Lep^ob/ob^* genetic background reduced body weight to a similar extent, while loss of leptin on the wild-type or *Rcan2* ^−/−^ genetic background increased body weight to a similar extent. This analysis indicates that *Rcan2* and leptin regulate body weight through different pathways.

Taken together, our current work has firmly established an important role of *Rcan2* in the regulation of food intake and body weight: firstly, the studies of *Rcan2* ^−/−^ mice showed that loss of *Rcan2* function significantly ameliorates age- and high-fat diet-induced obesity by causing a reduction of food intake; secondly, analysis of expression of *Rcan2* showed prominent expression in hypothalamic nuclei governing food intake and body weight; thirdly, fasting and refeeding experiment showed that *Rcan2-3* expression is up-regulated by fasting, not by leptin, in the hypothalamus, and loss of *Rcan2* significantly attenuates the hyperphagic response to starvation; finally and most importantly, using double-mutant (*Lep^ob/ob^ Rcan2* ^−/−^) mice, we were able to demonstrate that *Rcan2* and leptin regulate body weight through different pathways. Thus, our data indicate that there may be an *Rcan2*-dependent mechanism that regulates food intake and promotes weight gain through a leptin-independent pathway. These findings provide novel insights into the mechanisms of body weight regulation and should have important implications to studies on obesity in human population. The molecular basis of this putative pathway remains to be clarified. It is noteworthy that although *Rcan2* was originally identified as a T3-responsive gene [10], it was recently reported that T3 only regulates the expression of *Rcan2-3* [33], the splicing variant that is predominately expressed in the brain. However, studies of some seasonal animal species showed that hypothalamic T3 availability acts as a gatekeeper for seasonal control of body weight caused by alteration of food intake rather than energy expenditure [34], [35], although the downstream targets of T3 were not identified. In this study, we found that only *Rcan2-3* expression is up-regulated in the hypothalamus by fasting, which might be involved in the hyperphagic response to starvation. Future studies using in situ hybridization or PCR-analyses combined with micro-dissection technique will address the question of whether *Rcan2-3* rather than *Rcan2-1* is the molecule that is predominantly expressed in the hypothalamic nuclei and regulates food intake and body weight.

## Materials and Methods

### Generation of *Rcan2* ^−/−^ mice

The targeting vector was constructed by replacing the sequence corresponding to exon 4, which is used by *Rcan2-1* and *Rcan2-3,* with the *LacZ/Neo* cassette (Fig. S1a). In addition, a diphtheria toxin A expression cassette for negative selection was attached to the 3’ end of the *Rcan2* sequence in the targeting vector. The linearized vector was electroporated into 129Sv embryonic stem cells. A clone that had undergone the expected recombination was injected into blastocysts from C57BL/6J (B6) mice to obtain chimeric mice. The chimeric mice were then crossed with (B6 × CBA) mice. To transfer the *Rcan2* mutation onto a B6 genetic background, we used the mice produced in the latter cross to initiate repeated backcrosses with B6 mice to produce a B6-*Rcan2* ^+/−^ partial congenic strain. The *Rcan2* locus is located on chromosome 17. To limit the extent of the 129Sv-derived region around the targeted locus, we adopted a modified “speed congenesis” strategy from the fourth (N4) to sixth (N6) backcrosses. Briefly, the heterozygous offspring of the N4 generation were screened with polymorphic markers on chromosome 17 to select the mouse with the shortest 129Sv-derived chromosomal segment. The selected N4 mouse was then backcrossed with B6 mice to produce N5 offspring, and the same screen was repeated and again at the N6 generation. At the N6 generation, a mouse that contained an approximately 10 Mb 129Sv-derived chromosomal segment between markers *D17Mit24* and *D17Mit108* was selected for later backcrosses. The backcross was continued up to 14 generations. All the experimental work was conducted with mice that derived from the intercross of N7 to N14 B6-*Rcan2* ^+/−^ partial congenic mice.

Genotyping was carried out by PCR analysis of DNA extracted from tail tissue. The primers used for genotyping are listed in Table S2.

### Animal experiments

Mice were maintained in a specific pathogen-free barrier facility at the Research Institute of Environmental Medicine, Nagoya University, under conditions of constant temperature (23°C), a 12-h light/12-h dark cycle, and *ad lib* access to normal chow diet (4% total calories derived from fat, 2,835 kcalkg^−1^) (Labo MR Breeder, Nihon Nosan Co., Kanagawa, Japan) or high-fat diet (56.7% total calories derived from fat, 5,076 kcalkg^−1^) (High-Fat Diet 32, Clea Japan Inc.) and distilled water, unless noted otherwise. All animal experiments were approved by the ethical committee for animal experiments of the institute, in accordance with the Guidelines for Animal Experimentation of the Japanese Association for Laboratory Animal Science. Mice were weaned at postnatal day 21 (P21), and housed with 2∼4 littermates of the same gender per cage. Body weight was measured between 16:00 and 18:00 h once per week.

To generate *Lep^ob/ob^* mice lacking *Rcan2*, N10 B6-*Rcan2* ^+/−^ mice and *Lep^ob/+^* mice were crossed to produce the double heterozygote *Lep^ob/+^ Rcan2* ^+/−^. These mice were intercrossed to produce double homozygotes. Genotyping of *Lep* alleles was carried out by PCR analysis with the primers listed in Table S2.

### Food intake and fecal output measurement

*Rcan2* ^−/−^ and wild-type male mice were housed individually in cages with a steel screen for at least 10 days before measurement of food intake was started. Consumption of normal chow diet (or high-fat diet) and body weight were then measured for 3 weeks. To estimate food consumption, the food in each cage dispenser plus food that was spilled on the floor of the cage was weighed. Stools were collected every 24 h, dried for 2 days and then weighed.

Apparent absorption efficiency (AAE) was calculated using the following equation:



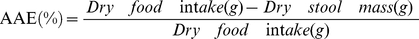



To examine the hyperphagic response to 24-hours fasting, the mice used for normal chow diet food intake measurement were selected for the analyses. After food intake measurement was terminated, the mice were weighed, and food was removed at 09:30 AM. Twenty four hours later, the mice were weighed and then given a weighted amount of normal chow diet, and the food intake was measured at 2, 4, 6, 10 and 24 h after refeeding. The body weight was also measured after the refeeding.

### Energy expenditure

Oxygen consumption and physical activity were measured using a Comprehensive Laboratory Animal Monitoring System (CLAMS, Columbus Instruments, Columbus, Ohio, USA) for 48 h. The mice were acclimatized for 60 h before measurements commenced.

### Glucose and insulin tolerance test

Glucose tolerance tests were performed on 15-h fasted mice. Animals were injected intra-peritoneally with D-glucose (15% solution; 1.5 g/kg of body weight), and blood glucose values were determined at 0, 30, 60, 90, and 150 min post-injection. Insulin tolerance tests were performed on 3-h fasted animals. Blood glucose values were measured immediately before and at 15, 30, 45 and 60 min after intra-peritoneal injection of porcine insulin (0.75 U/kg of body weight). Tail blood was taken and glucose levels were determined using a Medisafe glucose meter (TERUMO, Tokyo, Japan).

### X-gal staining

*Rcan2* ^−/−^ mice were anesthetized with Nembutal and transcardially perfused with 4% paraformaldehyde in 100 mM PBS for 10 min. Brains were dissected and 500 µm sections were prepared using brain matrices (Aster Industries, Pittsburgh, Pennsylvania, USA). The sections were fixed with 4% PFA in 100 mM PBS and 2 mM MgCl_2_ (pH 7.4) for 1 hour at 4°C, and then washed 3×10 min with PBS containing 0.1% Triton X-100 at room temperature. Staining was carried out at 37°C in PBS that contained 5 mM potassium ferricyanide, 5 mM potassium ferrocynide, 2 mM MgCl_2_, and 1 mg/ml X-gal.

### Real-time quantative RT-PCR

Total RNA was extracted from tissue samples using TriZol reagent (Invitrogen Japan, Tokyo, Japan). cDNA was synthesized from total RNA with a ReverTra Ace® qPCR RT kit (Toyobo, Osaka, Japan) and random hexamer primers. Fluorescence-based real-time quantitative RT-PCR was carried out using the Power SYBR® Green PCR kit and the ABI Prism 7000 Sequence Detection System (Applied Biosystems, Japan). Relative levels of mRNA were normalized against *β-actin,* then averaged and expressed as means ± s.e.m. The sequences of the primers used for quantitative PCR are listed in Table S2.

### Calcineurin phosphatase activity assay

Hypothalami were collected from 5-month-old male mice and lysed in calcineurin assay buffer (BioMol, Plymouth Meeting, PA). The Quantizyme Assay System AK-816 was used with 5 µg of protein according to the manufacturer's protocol. Calcineurin phosphatase activity was measured spectrophotometrically by detecting free phosphate released from the calcineurin-specific RII phosphopeptide.

### Statistical analysis

Data are expressed as means ± s.e.m. Two-tailed Student's *t* tests or one-way analysis of variance (ANOVA) were used to identify statistically significant differences (P<0.05).

## Supporting Information

Table S1 The ratios of mean body weights in age-matched mutant and wild-type mice
(0.05 MB DOC)

Table S2 Primers used for mouse genotyping and quantitative real-time PCR
(0.04 MB DOC)

Figure S1 Expression of oxidative phosphorylation-related genes. (A) Expression of oxidative phosphorylation-related genes in muscle. (B) Expression of oxidative phosphorylation-related genes in brown adipose tissue. Total RNA was isolated from muscle and BAT of 4-month-old males fed the normal chow diet and subjected to quantitative real-time PCR analysis using specific primers. mRNA expression levels are expressed relative to those of wild-type mice. Eight Rcan2+/+ and 9 Rcan2−/− males were analyzed. All values are given as mean ± s.e.m. Filled columns indicate Rcan2+/+ mice; open columns, Rcan2−/− mice.
(0.20 MB TIF)

Figure S2 Hypothalamic calcineurin activity in mice allowed to feed ad lib. Calcineurin activity in Rcan2−/− mice is expressed relative to that of Rcan2+/+ mice. Three males of each genotype were analyzed. All values are given as mean ± s.e.m. Filled columns indicate Rcan2+/+ mice; open columns indicate Rcan2−/− mice.
(0.09 MB TIF)

Figure S3 Expression of neuropeptide mRNAs in the hypothalamus. Total RNA was isolated from the hypothalami of 3-month-old male mice that were allowed to feed ad lib or were fasted for 24 h. The RNA was used for quantitative real-time PCR using primers specific for AgRP, NPY, POMC, MCH, prepro-orexin and β-actin (internal standard). Neuropeptide expression levels are expressed relative to those of ad lib fed wild-type mice. All values are given as mean ± s.e.m. Filled columns indicate Rcan2+/+ mice; open columns, Rcan2−/− mice. n  =  6 in each group. All values are given as mean ± s.e.m.
(0.21 MB TIF)

Figure S4 Expression of hypothalamic Rcan2-1 and Rcan2-3 mRNAs in the Lepob/ob mice. Total RNA was isolated from hypothalami of ad lib fed wild type or Lepob/ob male mice and subjected to quantitative real-time PCR analysis using primers specific for the Rcan2-1, Rcan2-3, NPY and β-actin (internal standard) genes. mRNA expression levels are expressed relative to those of ad lib fed wild type mice. Filled columns indicate wild type mice; open columns, Lepob/ob mice. n  = 5 in each group. All values are given as mean ± s.e.m. *: P<0.02.
(0.14 MB TIF)

## References

[1] Zimmet P, Alberti KG, Shaw J (2001). Global and societal implications of the diabetes epidemic.. Nature.

[2] Kahn BB, Flier JS (2000). Obesity and insulin resistance.. J Clin Invest.

[3] Eckel RH, Grundy SM, Zimmet PZ (2005). The metabolic syndrome.. Lancet.

[4] Spiegelman BM, Flier JS (2001). Obesity and the regulation of energy balance.. Cell.

[5] Lissner L, Heitmann BL (1995). Dietary fat and obesity: evidence from epidemiology.. Eur J Clin Nutr.

[6] Bray GA, Popkin BM (1998). Dietary fat intake does affect obesity!. Am J Clin Nutr.

[7] Lin S, Thomas TC, Storlien LH, Huang XF (2000). Development of high fat diet-induced obesity and leptin resistance in C57Bl/6J mice.. Int J Obes Relat Metab Disord.

[8] Buettner R, Scholmerich J, Bollheimer LC (2007). High-fat diets: modeling the metabolic disorders of human obesity in rodents.. Obesity (Silver Spring).

[9] Davies KJ, Ermak G, Rothermel BA, Pritchard M, Heitman J (2007). Renaming the DSCR1/Adapt78 gene family as RCAN: regulators of calcineurin.. Faseb J.

[10] Miyazaki T, Kanou Y, Murata Y, Ohmori S, Niwa T (1996). Molecular cloning of a novel thyroid hormone-responsive gene, ZAKI-4, in human skin fibroblasts.. J Biol Chem.

[11] Kingsbury TJ, Cunningham KW (2000). A conserved family of calcineurin regulators.. Genes Dev.

[12] Cao X, Kambe F, Miyazaki T, Sarkar D, Ohmori S (2002). Novel human ZAKI-4 isoforms: hormonal and tissue-specific regulation and function as calcineurin inhibitors.. Biochem J.

[13] Mizuno Y, Kanou Y, Rogatcheva M, Imai T, Refetoff S (2004). Genomic organization of mouse ZAKI-4 gene that encodes ZAKI-4 alpha and beta isoforms, endogenous calcineurin inhibitors, and changes in the expression of these isoforms by thyroid hormone in adult mouse brain and heart.. Eur J Endocrinol.

[14] Sanna B, Brandt EB, Kaiser RA, Pfluger P, Witt SA (2006). Modulatory calcineurin-interacting proteins 1 and 2 function as calcineurin facilitators in vivo.. Proc Natl Acad Sci U S A.

[15] Shima H, Pende M, Chen Y, Fumagalli S, Thomas G (1998). Disruption of the p70(s6k)/p85(s6k) gene reveals a small mouse phenotype and a new functional S6 kinase.. Embo J.

[16] Um SH, Frigerio F, Watanabe M, Picard F, Joaquin M (2004). Absence of S6K1 protects against age- and diet-induced obesity while enhancing insulin sensitivity.. Nature.

[17] Butler AA, Kozak LP (2010). A recurring problem with the analysis of energy expenditure in genetic models expressing lean and obese phenotypes.. Diabetes.

[18] O'Rahilly S (2009). Human genetics illuminates the paths to metabolic disease.. Nature.

[19] Himms-Hagen J (1997). On raising energy expenditure in ob/ob mice.. Science.

[20] Flier JS (2004). Obesity wars: molecular progress confronts an expanding epidemic.. Cell.

[21] Schwartz MW, Porte D (2005). Diabetes, obesity, and the brain.. Science.

[22] Morton GJ, Cummings DE, Baskin DG, Barsh GS, Schwartz MW (2006). Central nervous system control of food intake and body weight.. Nature.

[23] Elmquist JK, Elias CF, Saper CB (1999). From lesions to leptin: hypothalamic control of food intake and body weight.. Neuron.

[24] King BM (2006). The rise, fall, and resurrection of the ventromedial hypothalamus in the regulation of feeding behavior and body weight.. Physiol Behav.

[25] Porta S, Marti E, de la Luna S, Arbones ML (2007). Differential expression of members of the RCAN family of calcineurin regulators suggests selective functions for these proteins in the brain.. Eur J Neurosci.

[26] Goto S, Matsukado Y, Mihara Y, Inoue N, Miyamoto E (1986). The distribution of calcineurin in rat brain by light and electron microscopic immunohistochemistry and enzyme-immunoassay.. Brain Res.

[27] Sola C, Tusell JM, Serratosa J (1999). Comparative study of the distribution of calmodulin kinase II and calcineurin in the mouse brain.. J Neurosci Res.

[28] Zhang Y, Proenca R, Maffei M, Barone M, Leopold L (1994). Positional cloning of the mouse obese gene and its human homologue.. Nature.

[29] Considine RV, Sinha MK, Heiman ML, Kriauciunas A, Stephens TW (1996). Serum immunoreactive-leptin concentrations in normal-weight and obese humans.. N Engl J Med.

[30] Maffei M, Halaas J, Ravussin E, Pratley RE, Lee GH (1995). Leptin levels in human and rodent: measurement of plasma leptin and ob RNA in obese and weight-reduced subjects.. Nat Med.

[31] Qu D, Ludwig DS, Gammeltoft S, Piper M, Pelleymounter MA (1996). A role for melanin-concentrating hormone in the central regulation of feeding behaviour.. Nature.

[32] Wilding JP, Gilbey SG, Bailey CJ, Batt RA, Williams G (1993). Increased neuropeptide-Y messenger ribonucleic acid (mRNA) and decreased neurotensin mRNA in the hypothalamus of the obese (ob/ob) mouse.. Endocrinology.

[33] Cao X, Kambe F, Moeller LC, Refetoff S, Seo H (2005). Thyroid hormone induces rapid activation of Akt/protein kinase B-mammalian target of rapamycin-p70S6K cascade through phosphatidylinositol 3-kinase in human fibroblasts.. Mol Endocrinol.

[34] Ebling FJ, Barrett P (2008). The regulation of seasonal changes in food intake and body weight.. J Neuroendocrinol.

[35] Morgan PJ, Ross AW, Mercer JG, Barrett P (2006). What can we learn from seasonal animals about the regulation of energy balance?. Prog Brain Res.

