# Supplementary material for: Inactivation of the Rcan2 Gene in Mice Ameliorates the Age- and Diet-Induced Obesity by Causing a Reduction in Food Intake
Source: PLoS One. 2011 Jan 27;6(1):e14605. doi: 10.1371/journal.pone.0014605 (PMC3029291; doi:10.1371/journal.pone.0014605)
Supplement: Table S1 — The ratios of mean body weights in age-matched mutant and wild-type mice (0.05 MB DOC) [file pone.0014605.s001.doc]

| sex | genotype | Age (weeks) | | | | | | | | | | | | | | | | | |
| --- | --- | --- | --- | --- | --- | --- | --- | --- | --- | --- | --- | --- | --- | --- | --- | --- | --- | --- | --- |
|  |  | 3 | 4 | 5 | 6 | 7 | 8 | 9 | 10 | 11 | 12 | 13 | 14 | 15 | 16 | 17 | 18 | 19 | 20 |
| **♂** | *Rcan2*^−/−^ vs *Rcan2*^+/+^ | 0.83 | 0.80 | 0.84 | 0.89 | 0.91 | 0.92 | 0.92 | 0.92 | 0.92 | 0.91 | 0.91 | 0.91 | 0.90 | 0.89 | 0.88 | 0.87 | 0.87 | 0.87 |
|  | *double mutant* vs *Lep*^ob/ob^ | 0.98 | 0.88 | 0.84 | 0.86 | 0.86 | 0.88 | 0.88 | 0.89 | 0.88 | 0.89 | 0.89 | 0.88 | 0.88 | 0.88 | 0.88 | 0.87 | 0.87 | 0.86 |
|  |  |  |  |  |  |  |  |  |  |  |  |  |  |  |  |  |  |  |  |
|  | *Lep*^ob/ob^ vs *Rcan2*^+/+^ | 1.02 | 1.08 | 1.22 | 1.47 | 1.62 | 1.72 | 1.78 | 1.82 | 1.88 | 1.89 | 1.91 | 1.93 | 1.95 | 1.95 | 1.95 | 1.96 | 1.96 | 1.98 |
|  | *double mutant* vs *Rcan2*^−/−^ | 1.20 | 1.20 | 1.22 | 1.41 | 1.55 | 1.65 | 1.72 | 1.77 | 1.81 | 1.84 | 1.86 | 1.89 | 1.92 | 1.93 | 1.95 | 1.96 | 1.97 | 1.97 |
| **♀** | *Rcan2*^−/−^ vs *Rcan2*^+/+^ | 0.86 | 0.87 | 0.90 | 0.92 | 0.92 | 0.92 | 0.91 | 0.90 | 0.93 | 0.92 | 0.94 | 0.93 | 0.92 | 0.93 | 0.93 | 0.91 | 0.93 | 0.93 |
|  | *double mutant* vs  *Lep*^ob/ob^ | 0.97 | 0.93 | 0.89 | 0.89 | 0.91 | 0.91 | 0.91 | 0.92 | 0.92 | 0.92 | 0.93 | 0.93 | 0.92 | ND | ND | ND | ND | ND |
|  |  |  |  |  |  |  |  |  |  |  |  |  |  |  |  |  |  |  |  |
|  | *Lep*^ob/ob^ vs *Rcan2*^+/+^ | 1.05 | 1.19 | 1.36 | 1.64 | 1.88 | 2.02 | 2.12 | 2.17 | 2.30 | 2.34 | 2.40 | 2.40 | 2.41 | ND | ND | ND | ND | ND |
|  | *double mutant* vs *Rcan2*^−/−^ | 1.18 | 1.27 | 1.34 | 1.58 | 1.86 | 2.02 | 2.12 | 2.21 | 2.28 | 2.34 | 2.38 | 2.41 | 2.44 | ND | ND | ND | ND | ND |

**Table S1.** The ratios of mean body weights in age-matched mutant and wild-type mice

Ratios were calculated using the average body weights of the mice.

ND, Not determined.
