# Supplementary material for: Inactivation of the Rcan2 Gene in Mice Ameliorates the Age- and Diet-Induced Obesity by Causing a Reduction in Food Intake
Source: PLoS One. 2011 Jan 27;6(1):e14605. doi: 10.1371/journal.pone.0014605 (PMC3029291; doi:10.1371/journal.pone.0014605)
Supplement: Table S2 — Primers used for mouse genotyping and quantitative real-time PCR (0.04 MB DOC) [file pone.0014605.s002.doc]

**Table S2.** Primers used for mouse genotyping and quantitative real-time PCR

| PCR product name | Forward primer | Reverse primer | Product size |
| --- | --- | --- | --- |
| WT *Rcan2* allele | 5’-CCCGGATAGAGCTTCATGAGACTC-3’ | 5’-CCTACCAGAGACCAAACCACCAAG-3’ | 718 bp |
| Targeted *Rcan2* allele | 5’-GTCATAGCCGAATAGCCTCTCCAC-3’ | 5’-CCTACCAGAGACCAAACCACCAAG-3’ | 1005 bp |
| WT *Lep* allele | 5’-GCTGCACATGGAGAGGCCCAAC-3’ | 5’-CCAGCAGATGGAGGAGGTCTCG-3’ | 664 bp |
| Mutated *Lep* allele | 5’-GCTGCACATGGAGAGGCCCAAC-3’ | 5’-GCCAGCAGATGGAGGAGGTCTCA-3’ | 665 bp |
| Rcan2-1 | 5’-CCTGCAATGTTCACCAGTCTG-3’ | 5’-TCTGTCTCTGGGGTCTGGAC-3’ | 238 bp |
| Rcan2-3 | 5’-TCGCCTGTGTGGTGGATGTG-3’ | 5’-TCTGTCTCTGGGGTCTGGAC-3’ | 241 bp |
| AgRP | 5’-GTTCTGCTGTTGGCACTGCC-3’ | 5’-GAGAACGAGACTCGCGGTTC-3’ | 220 bp |
| NPY | 5’-CCGCCACGATGCTAGGTAAC-3’ | 5’-GCGGAGTAGTATCTGGCCATG-3’ | 160 bp |
| Prepro-orexin | 5’-GAGCTCCAGGCACCATGAAC-3’ | 5’-TCCCAGAGTCAGGATACCCG-3’ | 212 bp |
| MCH | 5’-GCAGAAAGATCCGTTGTCGC-3’ | 5’-CCATTCTCAGCTGGGAAGGC-3’ | 197 bp |
| POMC | 5’-CTAGGCCTGACACGTGGAAG-3’ | 5’-CTCCGTTGCCAGGAAACACG-3’ | 216 bp |
| Pgc-1 | 5’-CCGTGACCACTGACAACGAG-3’ | 5’-CCTGTGGGTGTGGTTTGCTG-3’ | 182 bp |
| mCPT1 | 5’-GCATGGAGACATTGGCCAAG-3’ | 5’-CGATACATGATCATGGCGTG-3’ | 247 bp |
| Ppar-δ | 5’-GAAAGAGGAAGTGGCCATGG-3’ | 5’-ATTCCATGTTGAGGCTGCCG-3’ | 174 bp |
| Ucp1 | 5’-CCTCTACGACTCAGTCCAAG-3’ | 5’-ATCCCATGCAGATGGCTCTG-3’ | 168 bp |
| Ucp3 | 5’-GACCCGATACATGAACGCTC-3’ | 5’-ATCACGTTCCAAGCTCCCAG-3’ | 141 bp |
| β-actin | 5’-TCGTTGCCGGTCCACAC-3’ | 5’-ATCGTCGCCCGCGAAG-3’ | 108 bp |
