# Supplementary figures and images for: Inactivation of the Rcan2 Gene in Mice Ameliorates the Age- and Diet-Induced Obesity by Causing a Reduction in Food Intake
Source: PLoS One. 2011 Jan 27;6(1):e14605. doi: 10.1371/journal.pone.0014605 (PMC3029291; doi:10.1371/journal.pone.0014605)

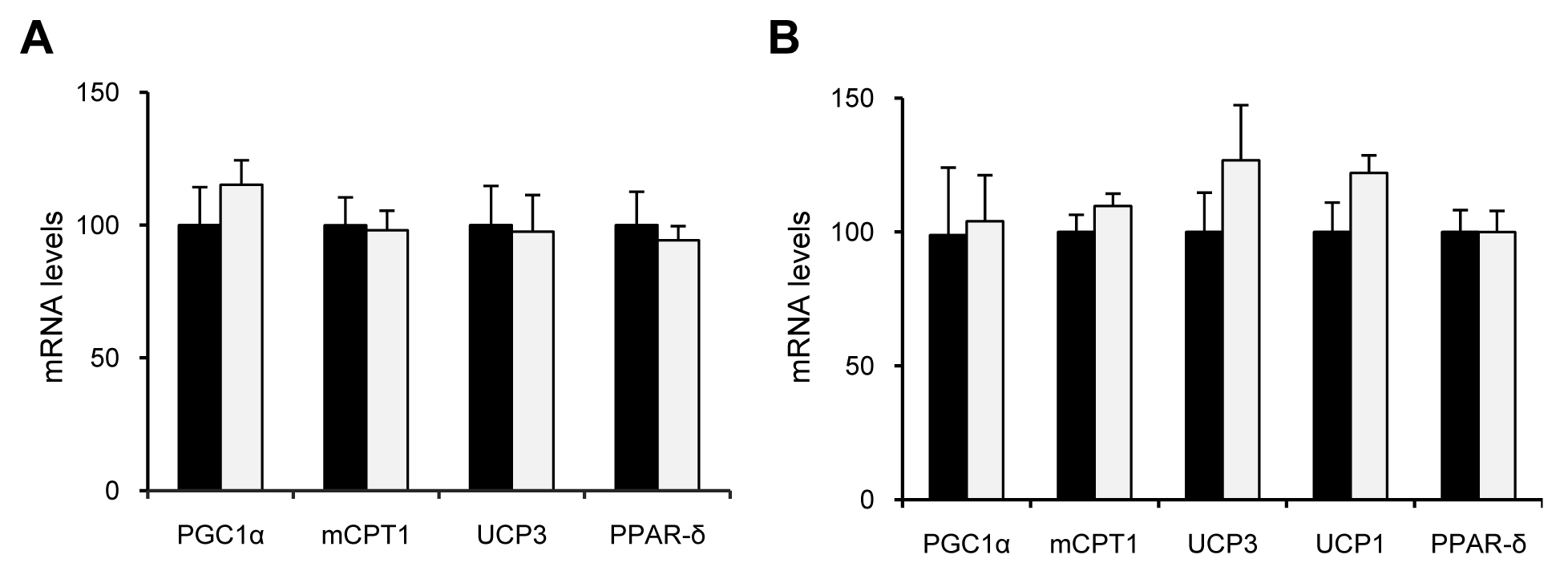

Supplement: Figure S1 — Expression of oxidative phosphorylation-related genes. (A) Expression of oxidative phosphorylation-related genes in muscle. (B) Expression of oxidative phosphorylation-related genes in brown adipose tissue. Total RNA was isolated from muscle and BAT of 4-month-old males fed the normal chow diet and subjected to quantitative real-time PCR analysis using specific primers. mRNA expression levels are expressed relative to those of wild-type mice. Eight Rcan2+/+ and 9 Rcan2−/− males were analyzed. All values are given as mean ± s.e.m. Filled columns indicate Rcan2+/+ mice; open columns, Rcan2−/− mice. (0.20 MB TIF) [file pone.0014605.s003.tif]

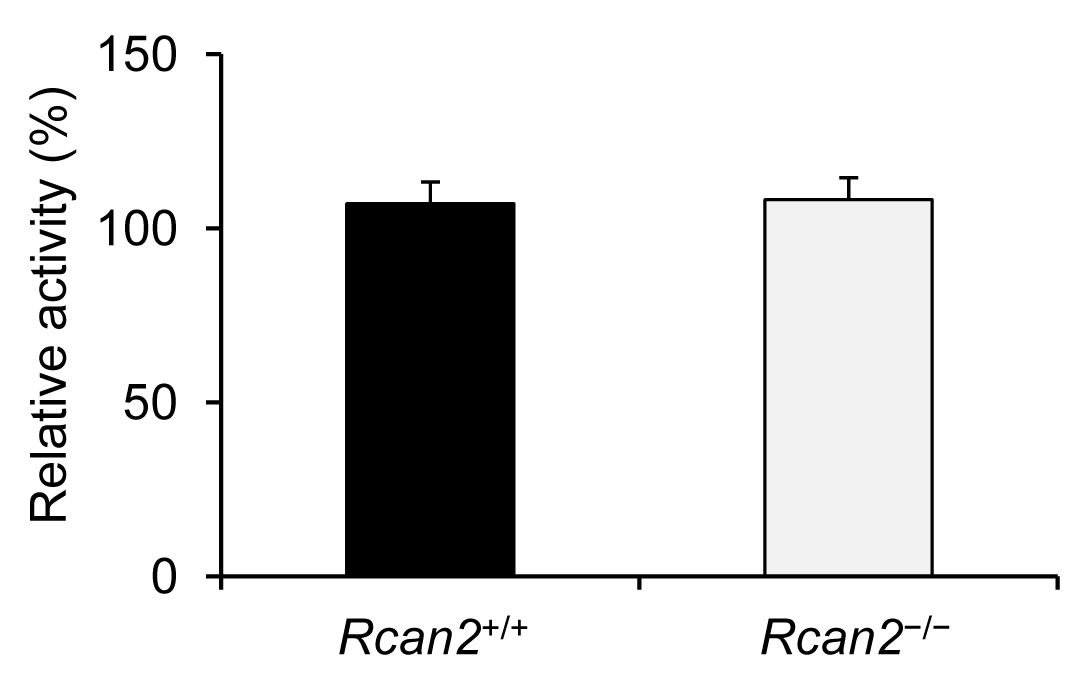

Supplement: Figure S2 — Hypothalamic calcineurin activity in mice allowed to feed ad lib. Calcineurin activity in Rcan2−/− mice is expressed relative to that of Rcan2+/+ mice. Three males of each genotype were analyzed. All values are given as mean ± s.e.m. Filled columns indicate Rcan2+/+ mice; open columns indicate Rcan2−/− mice. (0.09 MB TIF) [file pone.0014605.s004.tif]

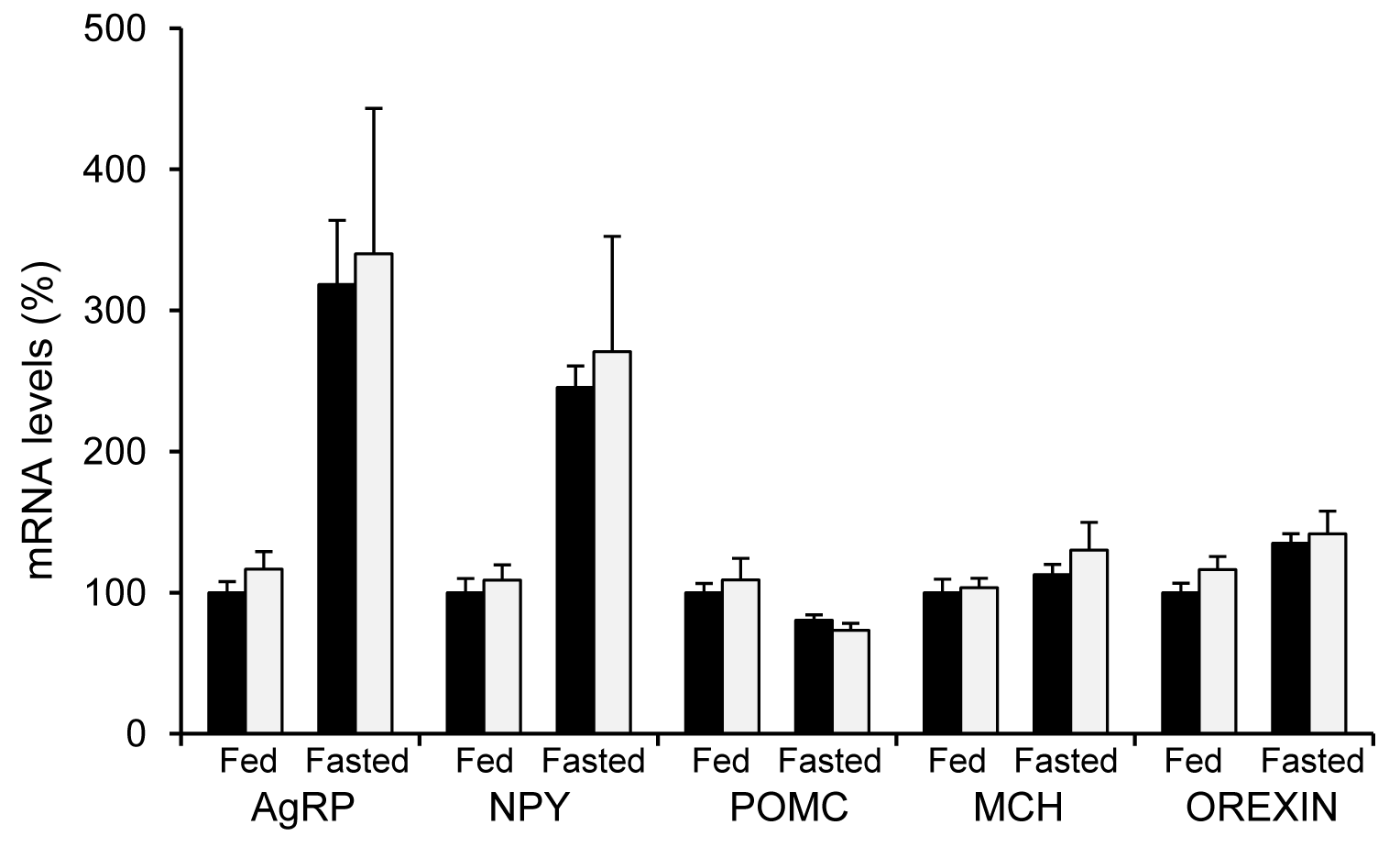

Supplement: Figure S3 — Expression of neuropeptide mRNAs in the hypothalamus. Total RNA was isolated from the hypothalami of 3-month-old male mice that were allowed to feed ad lib or were fasted for 24 h. The RNA was used for quantitative real-time PCR using primers specific for AgRP, NPY, POMC, MCH, prepro-orexin and β-actin (internal standard). Neuropeptide expression levels are expressed relative to those of ad lib fed wild-type mice. All values are given as mean ± s.e.m. Filled columns indicate Rcan2+/+ mice; open columns, Rcan2−/− mice. n = 6 in each group. All values are given as mean ± s.e.m. (0.21 MB TIF) [file pone.0014605.s005.tif]

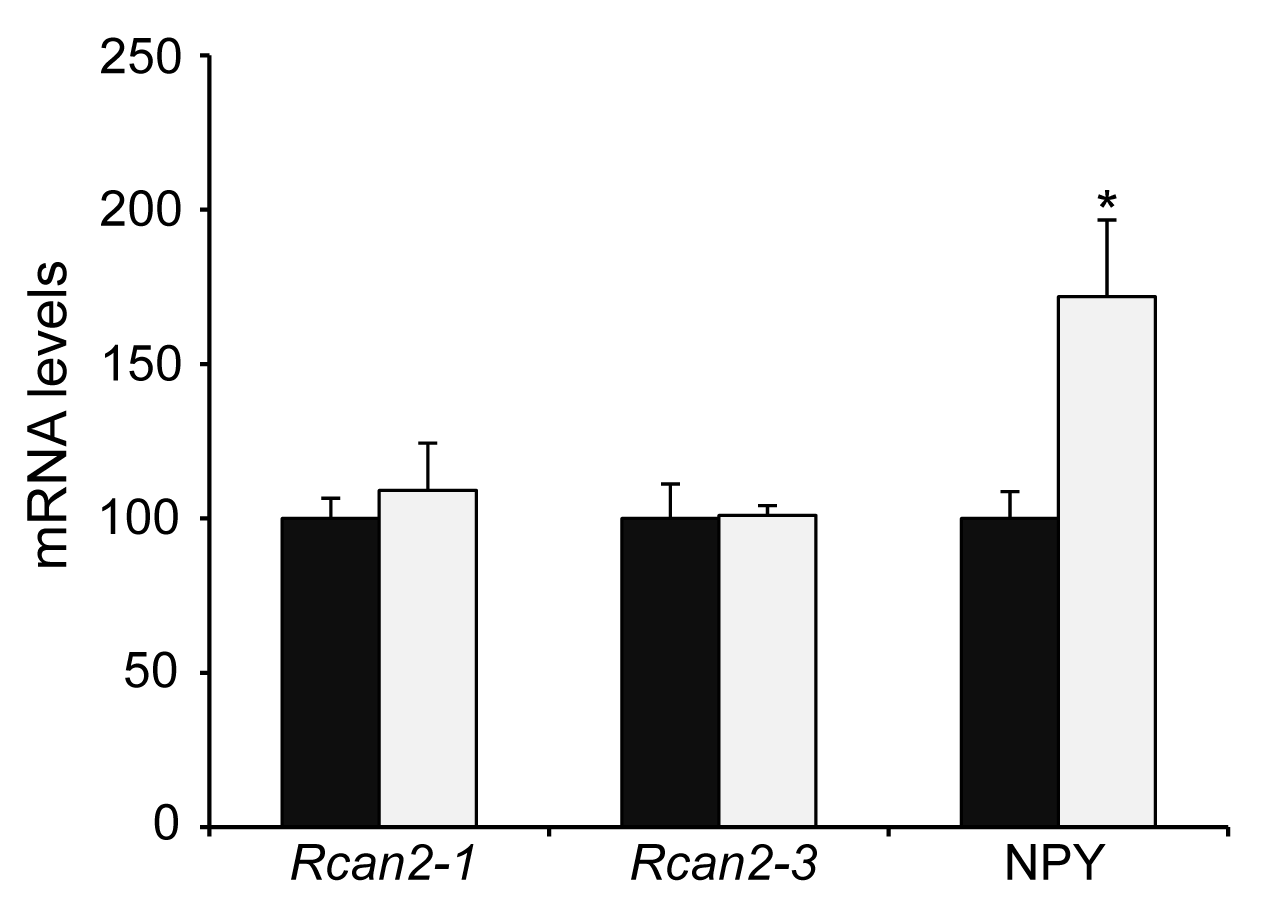

Supplement: Figure S4 — Expression of hypothalamic Rcan2-1 and Rcan2-3 mRNAs in the Lepob/ob mice. Total RNA was isolated from hypothalami of ad lib fed wild type or Lepob/ob male mice and subjected to quantitative real-time PCR analysis using primers specific for the Rcan2-1, Rcan2-3, NPY and β-actin (internal standard) genes. mRNA expression levels are expressed relative to those of ad lib fed wild type mice. Filled columns indicate wild type mice; open columns, Lepob/ob mice. n = 5 in each group. All values are given as mean ± s.e.m. *: P<0.02. (0.14 MB TIF) [file pone.0014605.s006.tif]
